# Smart Watches Lack Skin Smarts: Current and Future Dermatologic Applications in Device Metrics

**DOI:** 10.7759/cureus.55273

**Published:** 2024-02-29

**Authors:** Troy A Black, Mariya George, Morgan A Rousseau, Rashid M Rashid

**Affiliations:** 1 Dermatology, UTHealth Houston (University of Texas Health Science Center at Houston) McGovern Medical School, Houston, USA; 2 Internal Medicine, UTHealth Houston (University of Texas Health Science Center at Houston) McGovern Medical School, Houston, USA; 3 Dermatology, Mosaic Dermatology, Houston, USA

**Keywords:** wearables, ultraviolet, technology, smartwatch, prevention, sunscreen, dermatology, cancer

## Abstract

Introduction: Smartwatches have proven life-saving in medical specialties such as cardiology. Smartwatches actively warn us of arrhythmia risk and loud noise exposure. However, dermatologic health metrics are rarely monitored, and users are never alerted of potential skin health issues. Furthermore, the role of these devices within dermatology has not been evaluated in the literature. This study aims to analyze the current data points monitored by smartwatches and discuss potential adaptations to support dermatologic patient education and improve clinical management.

Methods: The top three smartwatches per global market share were identified and analyzed to determine the health data points they monitor and the alerts they provide. These data points were grouped and compared based on their corresponding body systems.

Results: Cardiovascular health comprises the highest percentage of data points collected with an average of 41% while dermatologic health averaged only 11%.

Conclusion: Dermatology is grossly underrepresented in current smartwatch devices. There is an important need to expand the dermatologic health metrics tracked by adapting existing smartwatch technology. From proactive cancer prevention to disease-specific reactive interventions, smartwatches can play a significant role in improving dermatological health and reducing healthcare costs.

## Introduction

Wearable sensors in the form of smartwatches have made it increasingly possible to monitor multiple aspects of individual health. In 2021, year-over-year growth of smartwatch global market shipments recorded an increase of 24%, with the fourth quarter setting a record high of over 40 million units shipped [1]. This increase correlates with the public’s heightened interest in monitoring their health. Smartwatches have been proven to be both accurate and beneficial in the monitoring of a wide range of health conditions, including, but not limited to, epilepsy, falls, self-management of chronic disease, medication adherence, and cardiologic ailments [2-5]. For example, in a study assessing smartwatches' ability to accurately notify users of atrial fibrillation, 84% of irregular pulse notifications were concordant with atrial fibrillation. If left undetected, atrial fibrillation can lead to stroke or heart failure; these devices have proven life-saving. As the popularity of smartwatches continues to increase, so does the range of sensors and features included in these devices. Despite these advances, few dermatological health data points are monitored by current smartwatches and none are available for alerts. Acute, chronic, and psycho-social issues of dermatological health are scarcely represented. Therefore, smartwatches appear to lack smarts when it comes to dermatology. 

The World Health Organization (WHO) estimates that one in every three cancers diagnosed is skin cancer. Despite a variety of efforts to address risk factors, skin cancer rates have continued to steadily increase worldwide [6]. In addition to skin cancers, up to 64.5% of people suffer from at least one skin condition [7,8]. Smartwatches sit on the skin and are therefore exposed to the same environmental factors. In fact, total lifetime exposure to radiation from the sun could represent a significant skin cancer risk and further increase patient awareness of exposure levels. Given the alarming prevalence of skin cancer, early detection is paramount to reducing morbidity and mortality. Incorporating increased monitoring through smartwatches can serve as a crucial tool in identifying abnormalities at their nascent stages. Other exposures these devices could help to monitor include allergens, irritants, and extremes of humidity.

Having easy access to health data, such as heart rate and rhythm, seems to facilitate closer self-monitoring, increased patient engagement, and potentially improved preventative outcomes. As such, the expansion of smart features to additional healthcare fields is warranted. As the healthcare emphasis continues to expand from reactive to proactive care, the devices can be particularly critical in dermatology. In this manuscript, we present an assessment of the current health data points monitored by the three smartwatches with the largest global market shares and discuss future opportunities for dermatologic smartwatch integrations.

## Materials and methods

Study design

Global smartwatch market share data was collected from the website Counterpoint Research, with the top three companies per market share percentage being Apple, Samsung, and Huawei [1]. Notification capabilities and specific health data points monitored by these smartwatches were gathered from their manufacturer's websites. Data points assessed include blood pressure, electrocardiogram (ECG), exercise, heart rate, standing, steps, swimming, temperature, air quality, sunrise/sunset times, UV (ultraviolet) index, hand washing, ambient light, fall detection, sleep, body composition, calorie expenditure, environmental sound levels, meditation, mindfulness, menstrual cycle tracking, blood oxygen, respiratory rate, and the maximum rate of oxygen consumption during exercise (VO_2_ max). These data points were sorted into the following categories: cardiovascular, dermatology, infectious disease, neurology, nutrition, otolaryngology, psychiatry, reproductive, and respiratory (Table 1).

**Table 1:** Health data points and alerts sent for the Apple Watch Series 7, Samsung Galaxy Watch 4, and Huawei Watch 3 The health data points and alerts across the Apple Watch Series 7, Samsung Galaxy Watch 4, and Huawei Watch 3 are categorized by medical specialty. "X" denotes the watch included that monitors or alerts. Image credits: T. Austin Black, Mariya George, Morgan A. Rousseau.

	Apple Watch Series 7		Samsung Galaxy Watch 4		Huawei Watch 3	
	Monitors	Alerts	Monitors	Alerts	Monitors	Alerts
Cardiovascular						
Blood Pressure			X	X	X	
ECG	X	X	X	X		
Exercise	X	X	X		X	
Heart Rate	X	X	X	X	X	
Standing	X	X	X	X	X	X
Steps	X		X		X	
Swimming	X	X	X	X	X	X
Temperature					X	
Dermatology						
Air Quality	X					
Sunrise/Sunset Times	X		X		X	
UV Index	X		X			
Infectious Disease						
Hand Washing	X	X				
Neurology						
Ambient Light					X	
Fall Detection	X		X		X	
Sleep	X		X	X	X	
Walking Steadiness	X					
Nutrition						
Body Composition			X			
Calorie Expenditure	X		X		X	
Otolaryngology						
Environmental Sound Levels	X	X				
Psychiatry						
Meditation	X	X	X	X		
Mindfulness	X	X				
Reproductive						
Menstrual Cycle Tracking	X		X			
Respiratory						
Blood Oxygen	X	X	X	X	X	
Respiratory Rate	X					
VO_2_ Max			X		X	
Total	20	10	17	8	14	2

Inclusion and exclusion criteria

Smartwatch inclusion criteria required each smartwatch to measure a minimum of one health data point and be within the top three global smartwatches by market share. Smartwatches that were not within the top three global smartwatches by market share and did not measure a minimum of one health data point were excluded.

Statistical analysis

The percentage of total health data monitored within each category was assessed for each of the three watches in question. Excel was used to calculate the percentage of health data points represented by each respective category of the total number of health data points captured by that smartwatch.

## Results

Globally, the top three smartwatches per market share percentage are Apple (30%), Samsung (10%), and Huawei (8%). 

Apple Watch Series 7

In the Apple Watch Series 7 (Apple Inc., Cupertino, CA), 30% of health data collected are composed of cardiovascular metrics, 15% are dermatology-related metrics, 5% are infectious disease, 15% are neurology metrics, 5% are nutrition metrics, 5% are otolaryngology metrics, 10% are psychiatry metrics, 5% are reproductive metrics, and 10% are respiratory metrics. The Apple Watch Series 7 can monitor steps, sleep, walking steadiness, calorie expenditure, menstrual cycle, and respiratory rate. Additionally, the watch utilizes an enhanced accelerometer to detect sudden falls, with an option to alert emergency services. The Apple Watch Series 7 tracks and notifies users regarding hand washing, environmental sound levels, meditation, mindfulness, blood oxygen levels, heart rate, standing, swimming, and other exercise. It can also utilize the wearer’s heart rate and rhythm to record an ECG and alert the wearer of abnormalities. Regarding dermatology-related health features, the Apple Watch Series 7 monitors sunrise/sunset times, UV index, and air quality. 

Samsung Galaxy Watch 4

The Samsung Galaxy Watch 4 (Samsung Electric Co., Ltd., Seoul, South Korea) has the second largest market share; in that, 41.18% of health metrics monitored are cardiovascular metrics, 11.76% are dermatology-related metrics, 11.76% are neurology metrics, 11.76% are nutrition metrics, 5.88% are psychiatry metrics, 5.88% are reproductive metrics, and 11.76% are respiratory metrics. The Samsung Galaxy Watch 4 can monitor steps, exercise, peak oxygen uptake, menstrual cycle, body composition, and calorie expenditure. Additionally, the watch utilizes an enhanced accelerometer to detect sudden falls, with an option to alert emergency services. The Samsung Galaxy Watch 4 monitors and alerts users of sleep, meditation, blood oxygen level, blood pressure, body composition, and swimming, and can perform an ECG utilizing the wearer’s heart rate and rhythm, and alert of abnormalities. Body composition is used to describe the percentages of fat, bone, water, and muscle in human bodies. Regarding dermatology-related health features, the Samsung Galaxy Watch 4 monitors UV index. 

Huawei Watch 3

Finally, in the Huawei Watch 3 (Huawei Technologies Co., Ltd., Shenzhen, China), 50% of health data collected are composed of cardiovascular metrics, 7.14% are dermatology-related metrics, 21.43% are neurology metrics, 7.14% are nutrition metrics, and 14.29% are respiratory metrics. The Huawei Watch 3 can monitor blood pressure, exercise, heart rate, steps, temperature, blood oxygen levels, ambient light, sleep, sudden falls, and calorie expenditure. The watch monitors and sends notifications to users regarding standing and swimming. Regarding dermatology-related health features, the Huawei Watch 3 monitors sunrise/sunset times. 

## Discussion

Current technology

**Figure 1:**
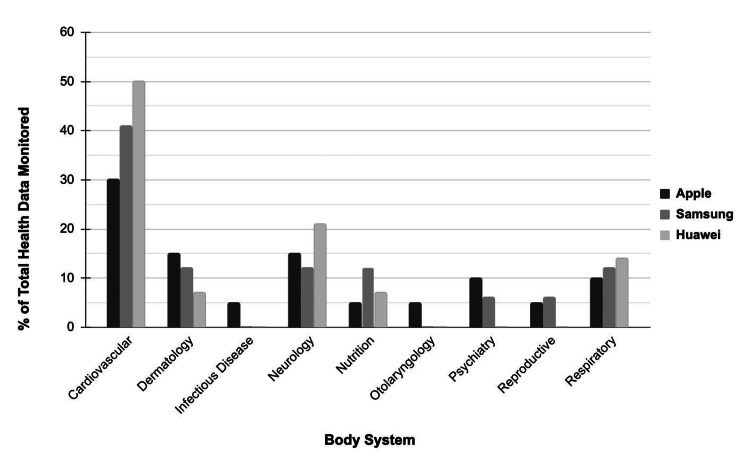
Percentage of total health data points monitored by body system Percentage analysis of the body system health metrics monitored by Apple Watch Series 7, Samsung Galaxy Watch 4, and Huawei Watch 3 categorized by body system. Image credits: T. Austin Black, Mariya George, Morgan A. Rousseau.

Smartwatches play a significant role in providing health data and affect healthcare outcomes. Most users purchase smartwatches to monitor fitness and health data [9]. Cardiology metrics have proven to be attractive to the public and industry, potentially due to the rapid and fatal nature of the outcomes. The top three smartwatches in the global market currently monitor and alert users of a variety of health data points. They will actively warn you about eminent health issues like loud noise danger and heart arrhythmia. However, only three of the 25 assessed health data points directly affect dermatologic health: air quality, UV index, and sunrise/sunset times with minor or no alerts. Of the top three smartwatches, the Apple Watch Series 7 monitors the most overall health and dermatology-related data points (Figure 1).

Air quality is currently reported only by the Apple Watch Series 7, but it could prove beneficial to expand this feature to other smartwatches. Exposure to highly polluted environments, combined with UV radiation, can cause photoaging and exacerbate skin diseases such as lupus erythematosus and dermatomyositis, and increase skin sensitivity in eczema and contact dermatitis patients [10]. Monitoring and alerting users of air quality can allow for the use of surface protection, such as sunscreen and antioxidants, and potentially limit exposure to poor air quality. 

Currently, the Apple and Samsung watches allow users to monitor the UV index. The Apple, Samsung, and Huawei watches also monitor sunrise and sunset times. UV radiation, a carcinogen emitted by the sun, is the most significant modifiable risk factor for melanoma and non-melanoma skin cancers [11]. UV exposure is correlated with skin wrinkling, elastosis, actinic keratoses, irregular pigmentation, telangiectasis, and the development of skin cancers [11-13]. Public awareness of the UV index has been shown to be associated with sun avoidance, and awareness of sunrise and sunset times could further increase sun-avoidant behaviors [11]. 

Future dermatologic adaptations

There is great potential for watches to monitor dermatology-related data and improve skin health with existing technology (Table 2). 

**Table 2:** Dermatological applications and benefits Dermatological applications in smartwatches and respective benefits. Image credits: T. Austin Black, Mariya George, Morgan A. Rousseau.

Application	Benefit
UV Index Alert	Help patients with xeroderma pigmentosum, porphyrias, photoallergy, lupus erythematosus, and other photosensitivity disorders
UV Exposure Risk Alert	Encourage sun-protective behavior (applying sunscreen, seeking shade, and getting regular skin checks)
UV Sensor	Provide quantitative, cumulative estimate of long-term UV exposure. Promote awareness of excessive UV exposure
Time Spent in Water	Helps guide users' sunscreen reapplication frequency
Face-Washing Monitor and Reminder	Improve patient compliance and clinical outcomes in those with acne vulgaris
Sleep Deprivation Alert	Inform users of inadequate sleep. Clinicians can use this data to educate patients on the impact of sleep on skin surface characteristics
Air Quality and Pollen Level Alerts	Promote the use of protective clothing, sunscreen with topical antioxidants, and the usage of indoor air purifiers/ventilators. Clinicians can use pollutant and pollen exposure data to guide diagnosis and treatment
Medication Reminder	Improve patient adherence to treatment plans
Pruritus Tracker	Track duration and frequency of itching in atopic dermatitis and other pruritic diseases. Clinicians can use data to guide management

For users with xeroderma pigmentosum, porphyrias, photoallergy, lupus erythematosus, and other photosensitivity disorders, smartwatch alerts of the UV index could allow users to take appropriate sun-protective measures and avoid sun exposure during high-risk periods [14]. While the UV index is currently monitored by some smartwatches, no devices provide alerts of this type. Alerts can be sent with a brief UV exposure risk statement (i.e., high cancer or photoaging risk) to educate users and mitigate the risk of tanning. Educating the public on the harmful effects of UV exposure could encourage sun-protective behavior such as applying sunscreen, seeking shade, and getting regular skin checks from dermatologists.

Additionally, cumulative UV exposure, particularly at a young age, is associated with a significantly increased risk for melanoma, basal cell carcinoma, and squamous cell carcinoma [15,16]. Currently, a small number of smartwatches on the market have the potential to monitor cumulative UV exposure through the utilization of UV and light intensity sensors. Implementing UV sensors into smartwatches, when combined with UV index data, could provide users with a quantitative, cumulative estimate of their long-term UV exposure [17]. A quantitative value of UV exposure across a user’s lifetime can promote awareness and encourage healthy behavior through an estimate of individual skin cancer risk. 

Currently, smartwatches have the ability to combine input from accelerometers and sensors which recognize the sound of water, to detect both hand washing and swimming. Using this technology, watches have the potential to monitor users’ time spent in water to guide sunscreen reapplication, especially following vigorous activity that could remove sunscreen. In a study conducted by Heerfordt et al., only 50% of beach-going patrons claimed to have applied sunblock during a local outing [18]. Of the 50%, only half claimed to have reapplied a second layer. Generally, sunscreen must be reapplied every 2 hours, with some sources citing even shorter reapplication times [19]. Proper sunscreen application is a known issue, but, when used correctly, can prevent sunburns and UV-induced skin lesions [20]. Compared to no reminder, studies providing participants with a physical reminder to reapply sunscreen have shown to increase reapplication rates [21,22]. Smartwatches have the potential to alert users to reapply sunscreen in a timely manner. 

In a clinical trial evaluating the effect of face washing on acne vulgaris, notable improvements in both open comedones and total noninflammatory lesions were observed in those who washed their face twice a day [23]. Currently, smartwatches can detect handwashing behaviors through the input of accelerometers and sensors which detect the sound of running water and squishing soap. These same sensors could be adapted to detect face-washing behaviors and remind wearers to perform this activity. In users with a variety of skin conditions, this could lead to improved patient compliance and clinical outcomes. 

The duration and quality of one’s sleep have been shown to significantly influence various skin characteristics [24]. Jang et al. found that after only one night of sleep deprivation (<4 hours per night), skin hydration, gloss, elasticity, and texture were significantly reduced (P < 0.01) [25]. In addition to sleep tracking, future smartwatches could provide users with sleep deprivation alerts to inform users of their sleep and skin health. Dermatologists can further use this data to educate patients regarding the effect of sleep deprivation and its impact on skin surface characteristics. 

Enabling smartwatches to alert users of air quality and pollen levels could be of great importance due to their significant associations with premature aging, atopic dermatitis, eczema, acne, psoriasis, acne, and skin cancers [26]. Prolonged exposure to pollutants such as cigarette smoke and UV radiation can lead to oxidative damage to the skin [27]. Smartwatches monitoring and alerting users of the air quality index and pollen could promote behaviors to avoid certain pollutants, the use of sunscreen with topical antioxidants, protective clothing, and the usage of indoor air purifiers and ventilators. Clinicians can use pollutant and pollen exposure data to aid in diagnosis, guide treatment, and educate patients. Further, many dermatological conditions, including those associated with pollutant exposure such as acne vulgaris, are treatable with the proper use of medications. In patients with such conditions, the implementation of customizable medication reminders on smartwatches could further guide treatment and improve outcomes. 

In patients with atopic dermatitis, a type I IgE-mediated hypersensitivity reaction, and various other pruritic diseases, itching and scratching are crucial factors in the maintenance of symptoms and can have a significant impact on the sufferer's quality of life [28]. Compounding this, many patients are not consciously aware of their scratching tendencies. As smartwatches have the capability to detect user movement, existing technology could be used to notify wearers when scratching movements occur, prompting them to discontinue the behavior. It could be further utilized to monitor scratching frequency in these patients, with the option to share recorded data with the patient's clinician as the duration of itching can provide clinically useful information regarding disease severity and management.

Limitations

Information for the Samsung Galaxy Watch 4 and Huawei Watch 3 was collected solely from data available on the respective company’s websites. The Apple Watch Series 7 was physically available, allowing a deeper insight into the range of health data monitored by the device. There is potential for incomplete reporting of all health data collected by the analyzed smartwatches due to the limited availability of information. Further, this analysis is not comprehensive of all possible health data that could be collected by the analyzed watches. Certain smartwatches allow for third-party applications to be installed which can greatly alter the device's performance and allow for additional health data points to be collected. In this paper, only the features included in the smartwatch's baseline software were analyzed.

## Conclusions

The public is increasingly interested in monitoring personal health and lifestyle data. The healthcare system evolution increasingly emphasizes both reactive and proactive care. Smartwatches today capture a wide range of health metrics, from heart rate to menstrual cycle. Despite smartwatches’ proximity to the skin and exposure to the same environment, this technology neglects dermatological health. Smartwatches are not skin-smart. With existing technology, there is great potential to expand the metrics tracked to benefit dermatologic health and influence clinical outcomes. Dermatology does not need to be out of sight or out of mind in the individual health monitoring world. In the future, smartwatches could implement these adaptations to support dermatological health, educate the public, and decrease the incidence of skin cancer.
